# A Summer Madrigal

**Published:** 1879-08

**Authors:** 


A SUMMER MADRIGAL—(*air "Pinafore"*)—

"Now the boy climbs up the trees,
And the verdant fruit doth seize;
And immediately the poison in his stomach camps,
And so do the fidgets and the colic and the cramps."

